# What the College of Nursing Has Accomplished

**Published:** 1919-11-22

**Authors:** Arthur Stanley

**Affiliations:** Chairman of the College of Nursing.


### WHAT THE COLLEGE OF NURSING HAS ACCOMPLISHED.

BY THE HON. SIR ARTHUR STANLEY, G.B.E., C.B., Chairman of the College of Nursing.

### A Letter to its Members.

IN view of the present crisis in the nursing profession I feel that some declaration of the policy of the College is desirable.

It is hardly necessary for me to point out to you that the whole aim of the College since its foundation has been to uplift, maintain, and protect the high standard essential in a profession which has for its object the nursing and care of suffering humanity.

The College is not encouraging the formation of a Trade Union for the Nursing profession, for, whilst it is essential that the economic conditions of the members should be carefully safeguarded, it is also essential that the priceless heritage given to the nurse in fulfilling in the highest sense the privilege of service to the sick should not be endangered or forgotten.

It must be remembered that whilst endeavouring to work with this ideal in view the College has at the same time laboured strenuously to improve and protect the economic conditions of its members, and I desire, therefore, to draw your attention to the work done by the College during the last four years.

**FOUR YEARS' COLLEGE WORK.**

**1. Headquarters have been established in London, including an Appointment and Enquiry Bureau, forming a centre for British nurses all over the world.**

**2. Centres have been formed in many of the large towns to deal with the educational and social work of the College, and to act as channels of communication between individual nurses in the country and country towns and the Council in London.**

**3. A Register of properly qualified Nurses has been compiled. (There are on this Register nearly 16,000 nurses.)**

**4. A Committee has been set up to deal with the curriculum of training-schools, post-graduate work, scholarships, and other educational matters.**

**5. A Committee has been set up to deal with questions of salaries, hours of work, etc. The first report of this Committee has been widely circulated throughout the Kingdom and has already produced marked improvement in the economic conditions under which Nurses work and live.**

**6. Striking success has been achieved in connection with State Registration of Nurses, the College having within its first four years of existence secured a pledge from the Government that they will bring in and pass a State Registration Bill without delay.**

**7. Scholarships have been endowed for the higher education of Nurses.**

**8 The Endowment Fund of the College has already reached the large amount of over £40,000.**

**9. The Tribute Fund has been established to help any Nurse in need or distress.**

**10. Members are receiving legal assistance free of cost.**

**11. A Cottage Home at Bonchurch, Isle of Wight, has been purchased and is being equipped, which, it is hoped, will be the first of many similar homes in different parts of the country.**

It is unnecessary for me to elaborate the enormous amount of detailed work and expenditure involved by the development of these schemes in four short years.

I feel that I need only ask most earnestly in the interests of those to whom you so willingly give of your best that you will in this time of unrest stand loyally and firmly by those who have no other object at heart but your interest and the uplifting and protection of the profession you love and serve so well.

I wish to assure you that what has been accomplished in so short a period in the face of such unjust and unreasonable opposition is only laying the foundation for much further work, which with your individual help and support I know will be accomplished in the near future.

Noisy opposition and unscrupulous attacks are best met by the quiet dignity of silence, but I feel that you, as a member of the College, have a right to expect from me the assurance that your interests are being fully safeguarded by those to whom you have confided them.

